# Determining the Rheological Parameters of Polymers Using Artificial Neural Networks

**DOI:** 10.3390/polym14193977

**Published:** 2022-09-23

**Authors:** Anton Chepurnenko

**Affiliations:** Strength of Materials Department, Faculty of Civil and Industrial Engineering, Don State Technical University, 344003 Rostov-on-Don, Russia; anton_chepurnenk@mail.ru; Tel.: +7-863-201-9136

**Keywords:** creep, relaxation, artificial neural networks, rheological parameters, polyvinyl chloride

## Abstract

Artificial neural networks have great prospects in solving the problems of predicting the properties of polymers. The purpose of this work was to study the possibility of using artificial neural networks to determine the rheological parameters of polymers from stress relaxation curves. The nonlinear Maxwell–Gurevich equation was used as the deformation law. The problem was solved in the MATLAB environment. The substantiation for the choice of the neural network input and output parameters was made. An algorithm for obtaining the data for neural network training was also proposed. Neural networks were trained on theoretical stress relaxation curves constructed with the Euler method. The value of the mean square error (MSE) was used as a criterion for the performance of the training. The constructed model of the artificial neural network was tested on the experimental relaxation curves of recycled polyvinyl chloride. The quality of the experimental curve approximation was quite good and was comparable with the standard methods for processing stress relaxation curves. Unlike the standard methods, when using artificial neural networks, no preliminary data smoothing was required. It is possible to use the proposed technique for processing not only relaxation curves, but also creep curves as well as processing creep tests not only in central tension, but also in bending, torsion and shear.

## 1. Introduction

Pronounced creep is characteristic for many polymers and composites based on them in addition to the elastic properties. For use in products for various purposes, it is important to be able to determine the rheological properties of polymer materials. In most existing techniques, the rheological parameters of polymers are determined from tests for creep under central tension [1,2,3,4]. The phenomenon of stress relaxation in polymers is interrelated with the phenomenon of creep and can be described by the same laws [5]; therefore, the rheological parameters of polymeric materials can also be determined from stress relaxation experiments [6,7,8,9].

One of the simplest rheological models, which is applied not only to polymers, but also to other materials such as concrete and wood, is the Maxwell–Thompson linear model. The creep strain $\varepsilon^{*}$ growth rate in this model under a uniaxial stress state is determined by the following expression [10]:

(1) $$\frac{\partial \varepsilon^{*}}{\partial t}=\frac{1}{nE}\left[\left(1-\frac{H}{E}\right)\sigma -H\varepsilon^{*}\right]$$

where σ is the stress, t is the time, E is the instant modulus of elasticity, H is the long modulus of elasticity and n is the relaxation time.

The product n⋅E is also called the relaxation viscosity $\eta^{*}$. In Equation (1), the viscosity is constant and does not depend on stress, which does not fully reflect the existing experimental data.

For many polymers, including polypropylene, polyvinyl chloride, high and low density polyethylene and polyurethane, a better agreement with the experimental data is provided by the nonlinear Maxwell–Gurevich equation, in which viscosity depends on stress [11,12,13,14,15]:

(2) $$\frac{\partial \varepsilon^{*}}{\partial t}=\frac{f^{*}}{\eta^{*}}$$

(3) $$f^{*}=\sigma -E_{\infty }\varepsilon^{*}$$

(4) $$\frac{1}{\eta^{*}}=\frac{1}{\eta_{0}^{*}}\exp\left(\frac{\left|f^{*}\right|}{m^{*}}\right)$$

Here, $f^{*}$ is the stress function, $E_{\infty }$ is the high elasticity modulus, $\eta_{0}^{*}$ is the initial relaxation viscosity and $m^{*}$ is the velocity modulus.

A more complex structure of the creep equation complicates the processing of the experimental data. The existing traditional methods for processing creep and relaxation curves based on the Maxwell–Gurevich equation [16,17] use numerical differentiation and require a sufficiently high quality of experimental curves.

Machine learning methods have great prospects in solving inverse problems, including the determination of the mechanical properties of materials. An artificial neural network (ANN) is one of the artificial intelligence methods that provides solutions for classification and regression problems. It is known as one of the best methods for data mining tasks. ANNs learn to predict output data using a set of attributes. The purpose of an ANN is to find solutions to problems in the same way that the human brain does [18].

Works [19,20,21] are examples of determining the properties of pavement materials with the help of an ANN. Papers [22,23] propose a method for determining the chemical, physical and mechanical properties of polymers based on their molecular structure using machine learning methods. In [24], artificial neural networks are used to predict the glass transition temperature of polymers based on their structure. Paper [25] proposes an optimized artificial intelligence model to predict the kerf quality characteristics in the laser cutting of basalt fibers reinforced with polymer composites. In [26], a new hybrid artificial intelligence approach is proposed to model the ultrasonic welding of a polymeric material blend. In [27], an ANN is used as one of the methods for predicting the properties of bistable morphing composites.

The possibilities of artificial neural networks are far from being exhausted.

The aim of this work was to study the possibility of using artificial neural networks to determine the rheological parameters of polymers based on the nonlinear Maxwell–Gurevich equation. The stress relaxation experiment was taken as a basis, but the approach could also be applied to the processing of the results of experiments on creep under central tension and other simple types of deformation such as bending, torsion and shear. The novelty of the proposed approach lay primarily in the choice of input parameters for the neural network as well as in the method of obtaining the data on which the neural network was then trained.

## 2. Materials and Methods

**Figure 1:**
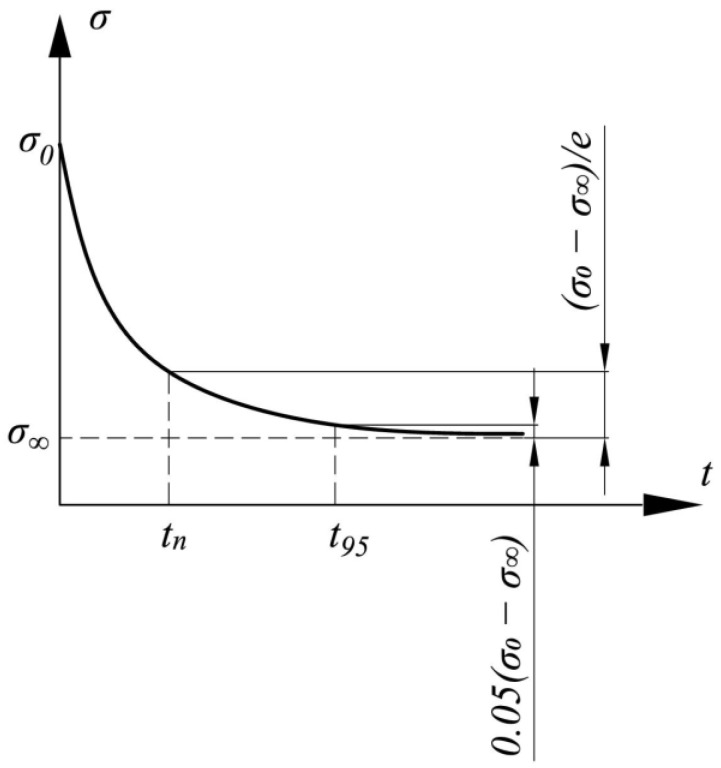
Typical stress relaxation curve.

Figure 1 shows a typical relaxation curve constructed with the Maxwell–Gurevich equation. Let us single out the characteristic points on this curve that were used as the input variables in the neural network: $\sigma_{0}$ is the stress at the initial moment of time; $\sigma_{\infty }$ is the stress at the end of the relaxation process (at *t*→∞); and $t_{n}$ is the time during which the stress drop Δ*σ* is:

(5) $$\Delta \sigma =\sigma \left(t_{n}\right)-\sigma_{0}=\left(\sigma_{0}-\sigma_{\infty }\right)\left(1-\frac{1}{e}\right)$$

Thus, $t_{95}$ was the time during which the stress drop was 95% of the maximum ($\sigma \left(t_{95}\right)-\sigma_{0}=0.95\cdot \left(\sigma_{\infty }-\sigma_{0}\right)$).

**Figure 2:**
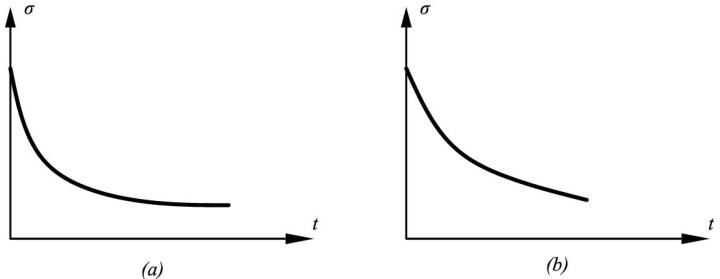
Good-quality (**a**) and bad-quality (**b**) stress relaxation curves.

The values $t_{n}$, $t_{95}$ and $\sigma_{\infty }$ can be determined from the experimental relaxation curve if it is of a sufficient quality. By the quality of the experimental curve, we mean here that in the experiment, one has waited for the stress relaxation curve to reach the horizontal asymptote. The tangent slope angle of the relaxation curve at the end point of the measurement should be close to zero (Figure 2).

The parameter $t_{n}$ was chosen as a characteristic value because, when using the Maxwell–Thompson linear creep law, it coincided with the relaxation time *n*.

Another input parameter of the neural network was the deformation *ε* at which the stress relaxation experiment was performed. If the value of the deformation is known, then the values of $\sigma_{0}$ and $\sigma_{\infty }$ can be used to easily determine the elastic modulus and the high elasticity modulus of the polymer, respectively. The modulus of elasticity was calculated by the formula:

(6) $$E=\frac{\sigma_{0}}{\varepsilon }$$

It is shown in [28] that, in the case of using the Maxwell–Gurevich equation, the relationship between stresses and strains at *t*→∞ has the form:

(7) $$\sigma_{\infty }=H\varepsilon \;$$

where $H=E\cdot E_{\infty }/\left(E+E_{\infty }\right)$ is the long modulus of elasticity.

Thus, if the values of *ε* and $\sigma_{\infty }$ are known, then it is not difficult to find the long modulus *H*. From the known values of *E* and *H*, one can then find $E_{\infty }$ using the formula:

(8) $$E_{\infty }=\frac{E\cdot H}{E-H}$$

Determining the values $m^{*}$ and $\eta_{0}^{*}$ is associated with further difficulties and an artificial neural network may be used for this. The input parameters of the network were the values *ε*, $\sigma_{0}$, $\sigma_{\infty }$, $t_{n}$ and $t_{95}$. At the output, the network should produce the parameters $m^{*}$ and $\eta^{*}$.

Network training was performed on the theoretical relaxation curves. For this, the possible ranges of change were selected in the modulus of elasticity $E\in \left[E_{1};E_{2}\right]$, high elasticity modulus $E_{\infty }\in \left[E_{\infty 1};E_{\infty 2}\right]$, velocity modulus $m^{*}\in \left[m_{1}^{*};m_{2}^{*}\right]$, initial relaxation viscosity $\eta_{0}^{*}\in \left[\eta_{01}^{*};\eta_{02}^{*}\right]$ and deformation $\varepsilon \in \left[\varepsilon_{1};\varepsilon_{2}\right]$. For each parameter in the specified ranges, the *m* values were generated and evenly spaced on the numerical axis. For $E,\;\;E_{\infty },\;\;m^{*}$ and $\eta_{0}^{*}$, we took 20 values; for ε, we took 3 standard values (1, 2 and 3%). Thus, the total number of options was 20^4^ × 3 = 480,000. For each option, a theoretical stress relaxation curve was constructed with the Euler method. The algorithm used for constructing the theoretical curve was as follows:

1. The long modulus of elasticity was calculated $H=E\cdot E_{\infty }/\left(E+E_{\infty }\right)$;
2. The stress $\sigma_{\infty }$ was calculated by Formula (7);
3. The maximum creep strain at *t*→∞ was determined by the formula:
  (9) $$\varepsilon_{max}^{*}=\frac{\sigma_{\infty }}{E_{\infty }}$$
4. The number of steps for the creep deformation $n_{\varepsilon }$ was set (we took it as equal to 200) and the step size was calculated:
  (10) $$\Delta \varepsilon^{*}=\frac{\varepsilon_{max}^{*}}{n_{\varepsilon }}$$
5. The creep strain growth rate at *t* = 0 was determined:
  (11) $$\frac{\partial \varepsilon^{*}}{\partial t}=\frac{\sigma_{0}}{\eta_{0}^{*}}\cdot \exp\left(\frac{\sigma_{0}}{m^{*}}\right)$$
6. The time step was calculated:
  (12) $$\Delta t=\frac{\Delta \varepsilon^{*}}{\frac{\partial \varepsilon^{*}}{\partial t}}$$
7. The creep strain at time t+Δt was then determined by the formula:
  (13) $$\varepsilon_{t+\Delta t}^{*}=\varepsilon_{t}^{*}+\frac{\partial \varepsilon^{*}}{\partial t}\Delta t$$
8. The stresses at time t+Δt were determined by the formula:
  (14) $$\sigma =E\left(\varepsilon -\varepsilon^{*}\right)$$
9. The strain growth rate was then calculated from the stresses using Formula (2).

Points (7)–(9) were then repeated until the creep strain reached the value $\varepsilon_{max}^{*}$. For each constructed curve, it was possible to determine the values $t_{n}$ and $t_{95}$. Two data arrays were then formed—*input* 5 × 480,000 in size and *target* 2 × 480,000 in size—on which the neural network was trained. The *input* array columns contained the values $\varepsilon ,\;\sigma_{0},\;\;\sigma_{\infty },\;t_{n}$ and $t_{95}$ for each calculated option. The *target* array columns contained the corresponding $m^{*}$ and $\eta_{0}^{*}$ values.

**Figure 3:**
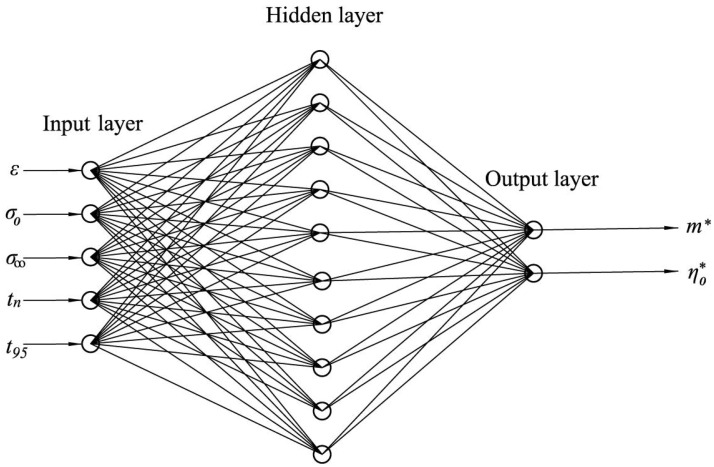
Neural network architecture.

The implementation of the neural network was made in the MATLAB environment. A feed-forward backpropagation network with one layer of hidden neurons was chosen as the network type. The number of hidden neurons varied from 10 to 14. The network architecture is shown in Figure 3. The input layer had 5 neurons, according to the number of input parameters. Each of the neurons of the input layer was connected to the neurons of the hidden layer by synapses with weights $w_{i}$. The neurons in the hidden layer transformed the signals coming from the input layer using an activation function. We used TANSIG (the hyperbolic tangent sigmoid transfer function) as an activation function in MATLAB. From the neurons of the hidden layer, the converted signal went to the neurons of the output layer. The neural network training process was the adjustment of the weights of the synapses [18].

The Levenberg–Marquardt method was used to adjust the weights of the network. There were four main functions for the evaluation of the training performance in MATLAB:

1. MSE (mean square error):
  (15) $$MSE=\frac{1}{n_{s}}\overset{n_{s}}{\underset{i=1}{\sum }}{\left(d_{i}-y_{i}\right)}^{2}$$
  where $y_{i}$ is the current value of the variable at the output of the network, $d_{i}$ is the target value and $n_{s}$ is the total number of output values for the considered sample.
2. MSEREG (mean squared error with regularization performance function). It measured the network performance as the weight sum of two factors: the mean squared error and the mean squared weight and bias values.
3. SSE (sum squared error):
  (16) $$SSE=\overset{n_{s}}{\underset{i=1}{\sum }}{\left(d_{i}-y_{i}\right)}^{2}$$
4. MAE (mean absolute error):
  (17) $$MAE=\frac{1}{n_{s}}\overset{n_{s}}{\underset{i=1}{\sum }}\left|d_{i}-y_{i}\right|$$

We chose the value of the MSE as the criterion for the training performance.

## 3. Results

An approbation of the technique for relaxation curve processing was carried out on the relaxation curves of the recycled polyvinyl chloride presented in [29]. In this work, the tests were carried out at a level of deformation of ε = 3%. The temperature in the experiment *T* changed from 20 to 70 °C with a step of 10 °C. The dependence of stresses on the time at different temperatures is shown in Table 1.

When training the neural network, the range of change in the elastic modulus *E* was taken from 400 to 4000 MPa; in the high elasticity modulus $E_{\infty }$, the range was from 0.05·*E* to 4·*E*, the velocity modulus $m^{*}$ varied from 1 to 15 MPa and the initial relaxation viscosity varied from 10^6^ to 10^8^ MPa·s. The generated data were randomly divided into three parts: training; validation; and testing in proportions of 70%, 15% and 15%.

**Figure 4:**
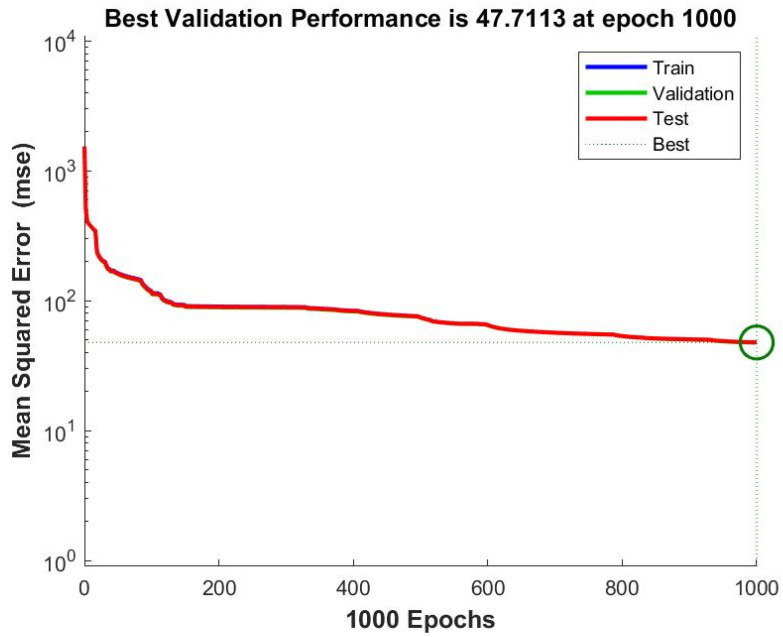
Neural net training performance graph.

**Figure 5:**
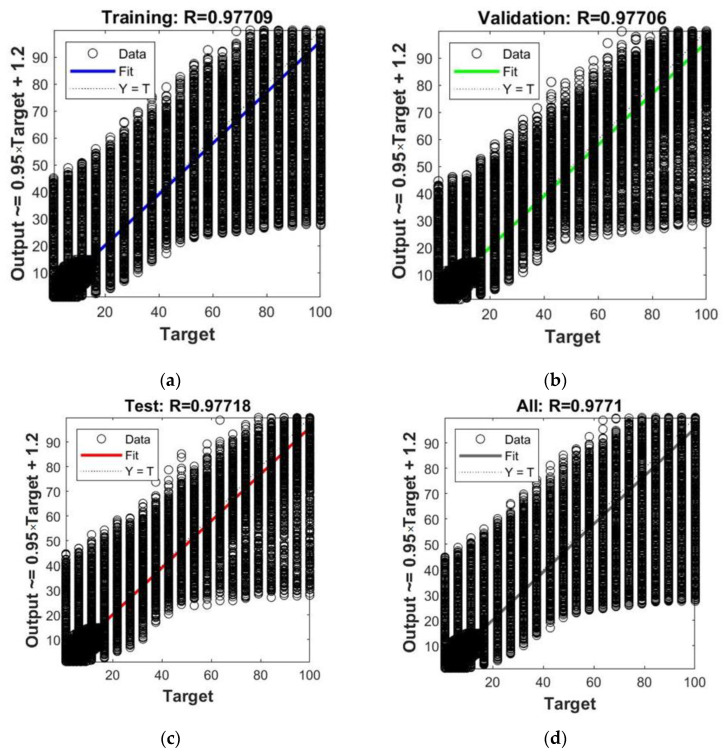
Regression charts for model with 12 hidden neurons: (**a**) Training sample; (**b**) Validation sample; (**c**) Test sample; (**d**) Full sample.

The optimal number of neurons in the hidden layer turned out to be 12. The neural net training performance graph is shown in Figure 4. When using the network with 12 neurons in the hidden layer, the best validation performance was 47.71 after 1000 iterations. Due to the large amount of data generated for training, the mean square errors for the samples “Train”, “Validation” and “Test” were almost the same. A further increase in the number of neurons led to the overtraining of the model. The regression charts of the model with 12 neurons are shown in Figure 5. For several input parameter values, there was a rather large deviation between the output predicted and the target values, which could be explained by the large sample size and wide range of input parameters. The correlation coefficients *R* between the output and the target values averaged 0.977. A value of the correlation coefficient close to 1 was one of the indicators of the possibility of using the model in the forecasting process.

Table 2 presents the values of the elastic and rheological parameters of the recycled polyvinyl chloride obtained using the proposed methodology at various temperatures.

**Figure 6:**
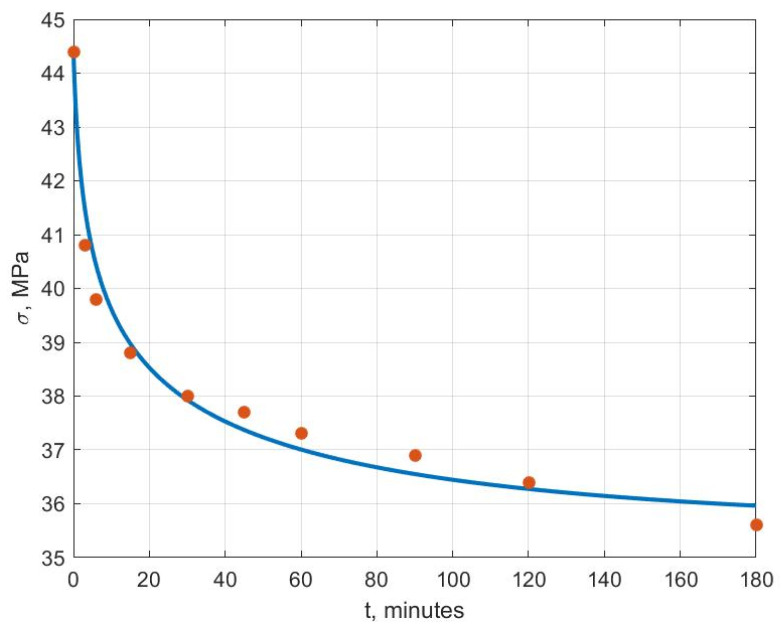
Stress relaxation curve at T=20 °C.

**Figure 7:**
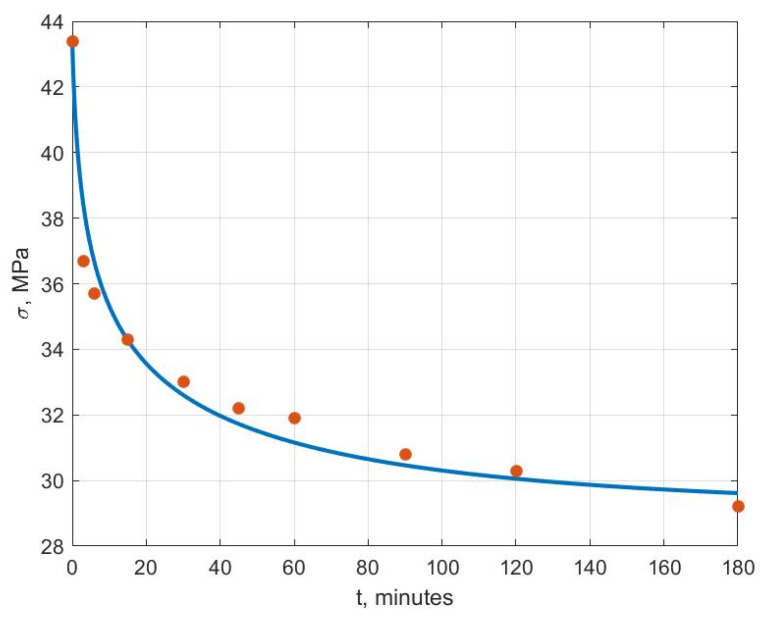
Stress relaxation curve at T=30 °C.

**Figure 8:**
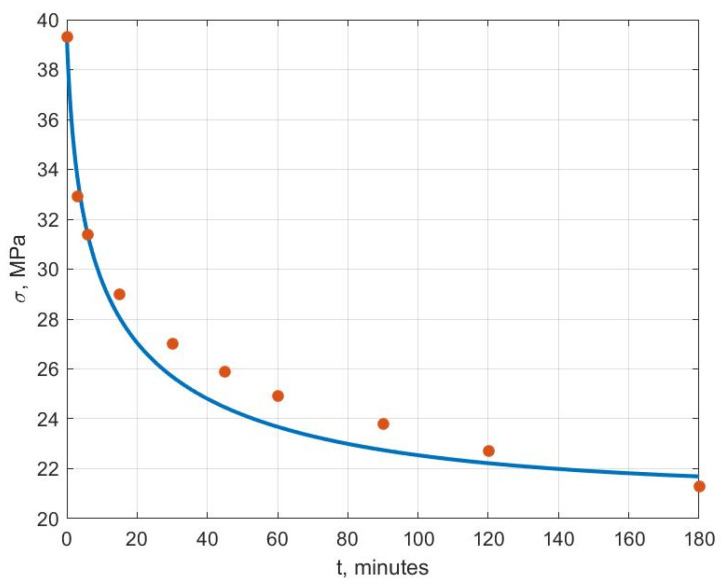
Stress relaxation curve at T=40 °C.

**Figure 9:**
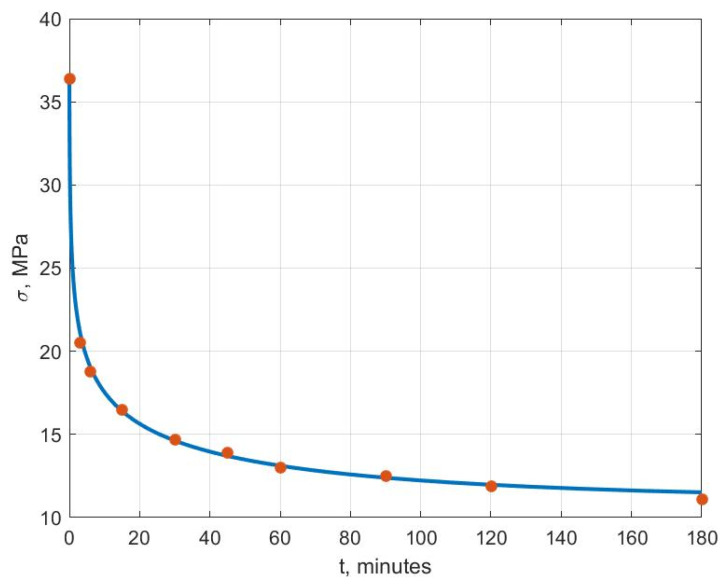
Stress relaxation curve at T=50 °C.

**Figure 10:**
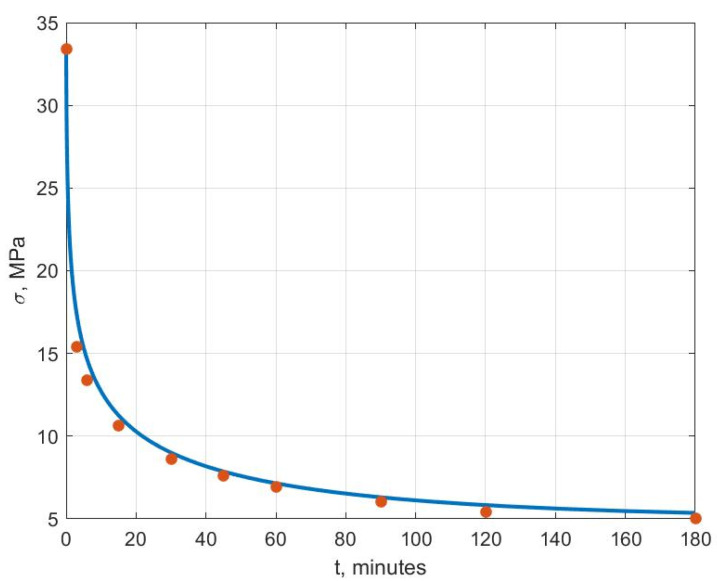
Stress relaxation curve at T=60 °C.

**Figure 11:**
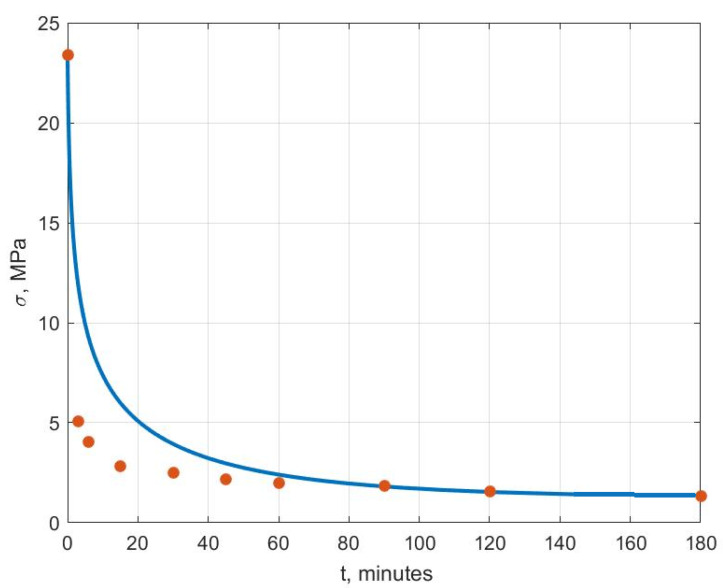
Stress relaxation curve at T=70 °C.

Figure 6, Figure 7, Figure 8, Figure 9, Figure 10 and Figure 11 show the theoretical stress relaxation curves plotted according to the data in Table 2. The experimental points are marked with round markers. For all temperatures except for 70 °C, there was a good agreement between the theoretical curves and the experimental data. At high temperatures, there was a strong decrease in the elastic and rheological characteristics of the polyvinyl chloride, which explained the not entirely good agreement of the results at 70 °C.

## 4. Discussion

In papers [16,17], the relaxation curves of the recycled polyvinyl chloride considered in this paper were processed earlier using a standard algorithm; in [30], nonlinear optimization methods were used to solve the same problem.

**Figure 12:**
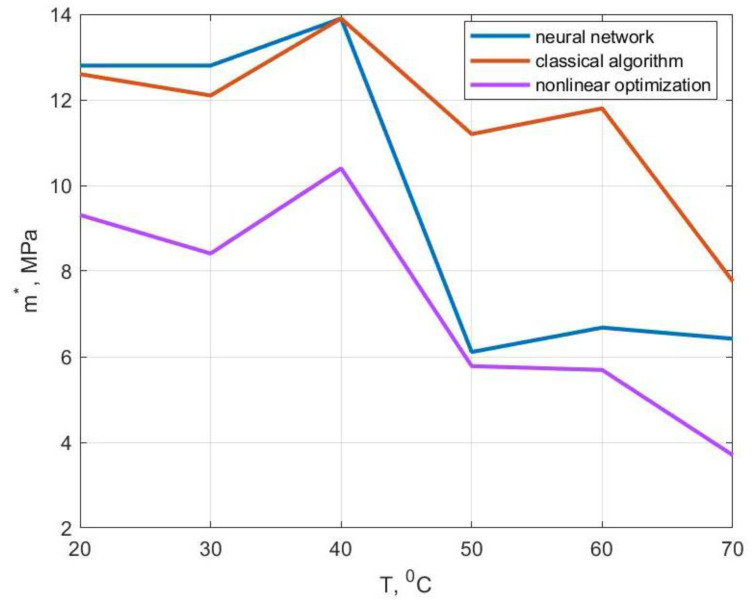
Comparison of the velocity modulus values obtained by different methods.

**Figure 13:**
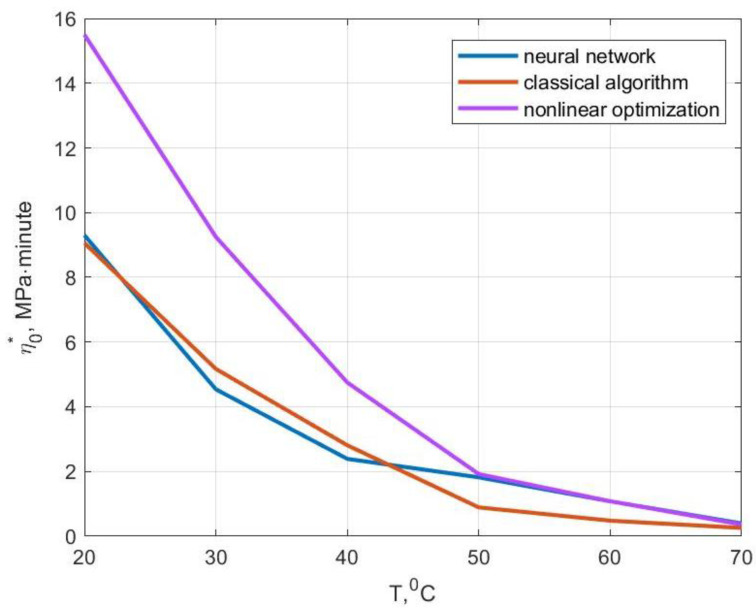
Comparison of the initial relaxation viscosity values obtained by different methods.

The values of the modulus of elasticity and the modulus of high elasticity obtained by us coincided with those given in [17] as machine learning methods were not used to determine them. A comparison of the values of the velocity modulus and the initial relaxation viscosity—obtained by the standard method using the methods of nonlinear optimization as well as using artificial neural networks—is shown in Figure 12 and Figure 13. For temperatures of 20, 30 and 40 °C, the values of the velocity modulus and initial relaxation viscosity obtained using a neural network were close to the results based on the classical algorithm. At temperatures of 50 and 60 °C, the solution based on the neural network was closer to the solution using nonlinear optimization methods. At 70 °C, the value of the velocity modulus obtained based on the artificial neural network was approximately in the middle between the results based on the other two methods and the relaxation viscosity was closer to the solution using nonlinear optimization methods.

Table 3 presents a comparison of the coefficients of determination $R^{2}$ showing the quality of the approximation using three methods for six considered curves.

Table 3 shows that the efficiency of the neural networks and the classical algorithm was approximately the same. However, the classical algorithm used the numerical differentiation of the function σ(t), which required a large number of points and the smoothing of the experimental curve. When using artificial neural networks, four characteristic points were sufficient and the smoothing of the experimental curve was not required. Nonlinear optimization methods are characterized by a higher quality of approximation; however, when using them, it is necessary to specify the initial approximation. If the real values of $m^{*}$ and $\eta_{0}^{*}$ are far from the initial approximation, then the solution may not be found. Nonlinear optimization methods can be used to refine a solution obtained by the classical algorithm or with the help of artificial neural networks.

Note that the proposed technique based on machine learning methods, with a small adjustment, could be used not only for processing the stress relaxation curves of polymers, but also for processing creep curves. It is also possible to obtain the rheological parameters of materials from tests not only for tension, but also for other simple types of deformations such as shear, bending and torsion.

## 5. Conclusions

The possibility of applying machine learning methods to solve the problem of determining the rheological parameters of polymers from stress relaxation curves has been shown. An artificial neural network model was built to determine the rheological parameters of recycled PVC at various temperatures. The optimal number of neurons in the hidden layer of the network was determined. The approbation of the model showed a good quality of approximation of the experimental curves at temperatures from 20 to 60 °C. The efficiency of the artificial neural networks in determining the rheological parameters of the polymers was comparable with the efficiency of traditional algorithms. However, in comparison with traditional algorithms, the smoothing of the experimental curves was not required. The proposed technique made it possible to determine the rheological parameters of the polymers not only from stress relaxation experiments, but also from experiments on creep as well as experiments on types of deformation such as torsion, shear and bending.

Our further research will be aimed at testing the creep of polymer samples in bending and building neural networks to process these experiments. Further research could also be devoted to the choice of the optimal neural network architecture and the most effective algorithms for training.

## Figures and Tables

**Table 1:** Time dependence of stresses at different temperatures.

Time, Minutes		0	3	6	15	30	45	60	90	120	180
σ, **MPa**	T, °C										
	20	44.4	40.8	39.8	38.8	38.0	37.7	37.3	36.9	36.4	35.6
	30	43.4	36.7	35.7	34.3	33.0	32.2	31.9	30.8	30.3	29.2
	40	39.3	32.9	31.4	29.0	27.0	25.9	24.9	23.8	22.7	21.3
	50	36.4	20.5	18.8	16.5	14.7	13.9	13.0	12.5	11.9	11.1
	60	33.4	15.4	13.4	10.6	8.61	7.63	6.95	6.02	5.42	5.05
	70	23.4	5.06	4.05	2.84	2.48	2.16	2.00	1.85	1.58	1.31

**Table 2:** Elastic and rheological parameters of recycled polyvinyl chloride at different temperatures.

T, °C	20	30	40	50	60	70
E, MPa	1480	1450	1310	1210	1113	780
$E_{\infty },$ MPa	5990	2970	1550	532	198	46.3
$m^{*}$, MPa	12.8	12.8	13.9	6.11	6.68	6.42
$\eta_{0}^{*},\;\mathrm{MPa}\cdot \mathrm{minute}$	9.3 × 10^5^	4.54 × 10^5^	2.39 × 10^5^	1.82 × 10^5^	1.08 × 10^5^	3.94 × 10^4^

**Table 3:** Comparison of $R^{2}$ determination coefficients using three methods for determining the rheological parameters.

*T*, °C		20	30	40	50	60	70
***R* ^2^**	Neural network	0.9772	0.9668	0.9693	0.9985	0.9899	0.7604
	Classical algorithm	0.9798	0.9712	0.9811	0.9421	0.9450	0.7796
	Nonlinear optimization	0.9918	0.9905	0.9909	0.9992	0.9994	0.9988

## Data Availability

Not applicable.
